# Traction force microscopy of engineered cardiac tissues

**DOI:** 10.1371/journal.pone.0194706

**Published:** 2018-03-28

**Authors:** Francesco Silvio Pasqualini, Ashutosh Agarwal, Blakely Bussie O'Connor, Qihan Liu, Sean P. Sheehy, Kevin Kit Parker

**Affiliations:** 1 Disease Biophysics Group, Wyss Institute for Biologically Inspired Engineering, Harvard University, Cambridge, MA, United States of America; 2 John A. Paulson School of Engineering and Applied Sciences, Harvard University, Cambridge, MA, United States of America; 3 Department of Biomedical Engineering, University of Miami, Miami, FL, United States of America; 4 Department of Pathology, University of Miami Miller School of Medicine, Miami, FL, United States of America; 5 Dr. John T. Macdonald Foundation Biomedical Nanotechnology Institute, Miami, FL, United States of America; The University of Akron, UNITED STATES

## Abstract

Cardiac tissue development and pathology have been shown to depend sensitively on microenvironmental mechanical factors, such as extracellular matrix stiffness, in both *in vivo* and *in vitro* systems. We present a novel quantitative approach to assess cardiac structure and function by extending the classical traction force microscopy technique to tissue-level preparations. Using this system, we investigated the relationship between contractile proficiency and metabolism in neonate rat ventricular myocytes (NRVM) cultured on gels with stiffness mimicking soft immature (1 kPa), normal healthy (13 kPa), and stiff diseased (90 kPa) cardiac microenvironments. We found that tissues engineered on the softest gels generated the least amount of stress and had the smallest work output. Conversely, cardiomyocytes in tissues engineered on healthy- and disease-mimicking gels generated significantly higher stresses, with the maximal contractile work measured in NRVM engineered on gels of normal stiffness. Interestingly, although tissues on soft gels exhibited poor stress generation and work production, their basal metabolic respiration rate was significantly more elevated than in other groups, suggesting a highly ineffective coupling between energy production and contractile work output. Our novel platform can thus be utilized to quantitatively assess the mechanotransduction pathways that initiate tissue-level structural and functional remodeling in response to substrate stiffness.

## Introduction

In the heart, striated cardiomyocytes seamlessly merge into two-dimensional layers that wrap around three-dimensional atria and ventricles. When single myocytes contract, the muscular layers compress and twist the heart chambers pumping blood into the body [1,2]. This exquisitely-controlled, multi-scale dynamic is difficult to study *in-vivo* as one must compromise the organ structure to isolate individual cardiomyocytes. Alternatively, the spatial complexity of the heart musculature may be reconstructed *in-vitro* using cardiac microphysiological systems (MPS)[3,4]. In these platforms, we engineer muscle cells and tissues to recapitulate native-like structure and assess their contractile function using a variety of techniques. For example, in isolated cells, traction force microscopy (TFM) is used to monitor the planar displacement of micro-beads and pillars embedded in the culture substrate as it deforms during cardiomyocyte shortening [5–9]. Instead, at the tissue-level, light microscopy is employed to track the bending motion of tissue-anchoring structures, such as cantilevers or posts [10–14]. Unfortunately, the sensitivity and specificity of these measurement techniques vary as a function of substrate mechanics, extracellular matrix (ECM) compositions, fluid dynamic interactions, and microscope properties. Ultimately, since cardiac MPS at different spatial scales use distinct assessment techniques, our ability to quantitatively study the multi-scale nature of the heart musculature *in-vitro* remains limited.

Additionally, the coupling of MPS-based structural-functional characterizations with metabolic assessments has remained challenging. We previously utilized traction force microscopy (TFM) to correlate contractile structure and function of isolated cardiomyocytes [7], as well as homogeneous [8] and heterogeneous [5] pairs of primary and stem cell-derived cardiomyocytes cultured on microenvironments with distinct mechanical properties. These studies indicated that physiological stiffness is necessary to obtain mature structural architecture and optimal force generation. Interestingly, in a separate study, we found that metabolism also modulates contractile structure and function [15]. Using an automated flux analyzer (Seahorse), we showed that engineered tissues assembled from neonate rat ventricular myocytes (NRVM) seeded on soft hydrogels retained a higher ability to initiate synthesis of adenosine triphosphate (ATP) than cells seeded on plastic culture dishes [16]. Unfortunately, it has remained difficult to investigate the relationship between metabolism, cytoskeleton structure, and contractile function, as multiscale platforms compatible with metabolic, structural, and functional profiling were missing.

Here, we present a mini tissue (mTissue) TFM platform to measure tractional forces in engineered cardiac tissues and asked whether changes in substrate stiffness affect energy production and utilization in primary cardiomyocytes. To achieve this, we microcontact printed mm-scale fibronectin islands on polyacrylamide (PA) gels with embedded fluorescent beads. We prepared the gels to mimic the stiffness of fetal (~1 kPa), adult physiological (~13 kPa) and adult pathological (~90 kPa) myocardium [7,8]. We then engineered anisotropic cardiac mTissues to assess the contractile structure and function. Finally, we cast PA gels with the same three levels of stiffness inside the multi-well plates of a commercially available Seahorse flux analyzer to correlate tissue metabolism, structure, and function. Our results suggest that the coupling between metabolism and contractile structure and function is weakest in cardiac tissues engineered on soft-substrate; peaks in tissues cultured on substrates of physiological stiffness; and degrades in stiffer substrates where elevated peak systolic stress fails to generate sufficient displacement.

## Results

### A tissue level TFM platform

**Fig 1:**
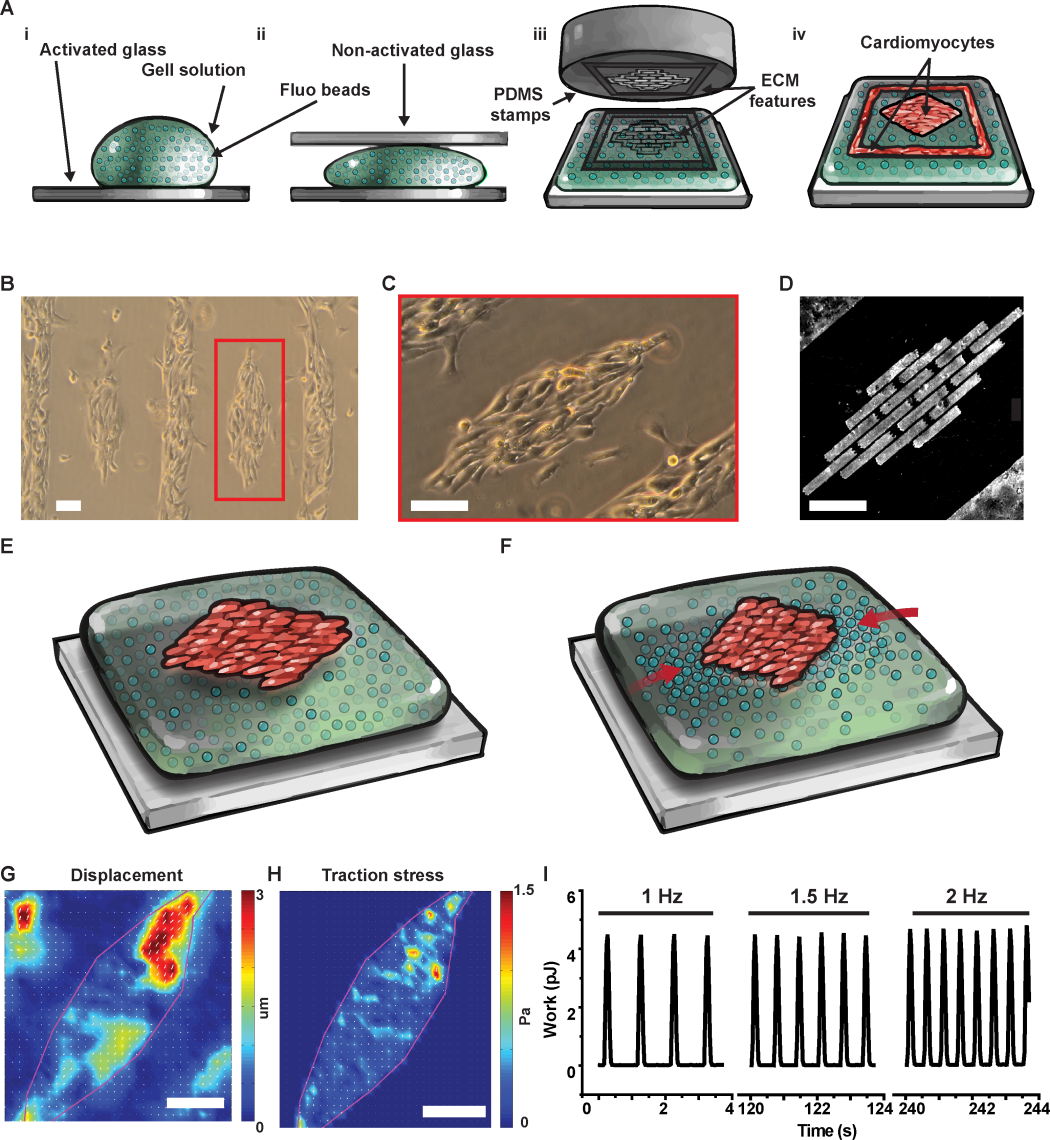
Micropatterned cardiac tissues on soft gels for traction force microscopy. (A) We cast PA gels (i) sandwiching the pre-polymer solution between activated and non-activated glass (ii) before stamping fibronectin (iii) to promote cell adhesion on the gel surface (iv). With this versatile method, we engineered neonate rat ventricular myocytes (NRVM) into diamond-shaped mini tissues (B) featuring cells aligned along the major axis of the diamond (C) thanks to a micro-contact printed brick-wall pattern of fibronectin (D). By tracking the displacement of fluorescent beads embedded in the in the soft gel during cell relaxation (E) and contraction (F), we used traction force microscopy to obtain displacement (G), and stress (H) maps at the tissue level. Importantly, since we could electrically stimulate the diamond-shaped tissues, we could measure displacement, stress, and contractile work as a function of beating frequency (I). Scale bars: 100 μm.

Cardiomyocytes are mechanosensitive cells that can significantly alter their structural and functional properties as a function of the rigidity of their microenvironment to measure contractile force *in-vitro*, gelatin, alginate, and PDMS have been used, but it is difficult to independently control the mechanical properties of the substrate and the geometry of cells using these materials [6]. Here, instead, we used the streptavidin/biotin chemistry to strongly bind custom-designed islands of fibronectin on thin PA gels of tunable stiffness. First, we cast thin PA gels with a nominal thickness of 80 μm by sandwiching a 20 μL drop of pre-polymer solution (Fig 1A, panel i) between glass coverslips (Fig 1A, panel ii). We tuned the mechanical properties of these gels by varying the acrylamide/bis concentration in the monomer solution; and bound ECM proteins to the gel surface by adding streptavidin-acrylamide to the mix before casting. Second, we micro-contact printed biotinylated-fibronectin (Fig 1A, panel iii) using stamps carrying two distinct features: *i)* a connected grid with horizontal and vertical spacing of 1 mm and 0.5 mm, respectively; and *ii)* isolated diamond-shaped islands (Fig 1A, panel iv) leading to contracting mini tissues 0.5 mm in length and 0.2 mm in width (Fig 1B). We speculated that the external grid provides mechanical support by reducing off-shape ECM deposition on soft gels and that cells growing in the grid may support the mTissues by providing pro-survival paracrine signals. Also, to obtain native-like laminar tissues (Fig 1C), we engineered diamond-shaped islands featuring a brick wall pattern with 100 μm long and 20 μm wide bricks separated by 5 μm sawtooth-shaped gaps (Fig 1D) [17].

Finally, to assess the contractile properties of the engineered tissues, we incorporated fluorescent beads (200 nm in diameter emitting at 560 nm) in the gel and used a confocal microscope to image bead displacement immediately below the tissue during several consecutive contraction cycles (Fig 1E and 1F). From the bead displacement field (Fig 1G), we calculated the cell-generated traction stress map using Fourier transform traction cytometry (FTTC; Fig 1G) [18]. Importantly, and differently from single cell and cell pair preparations, engineered mTissues can be electrically paced allowing for assessment of the contractile work output at various beating frequencies (Fig 1I). Together, these results demonstrate that our platform can be used to simulate the pathophysiological stiffening of the myocardium observed during development and disease, and assess the resulting structural and functional changes experienced by cardiomyocytes.

### Contractile structure and function of NRVM in the tissue TFM platform

**Fig 2:**
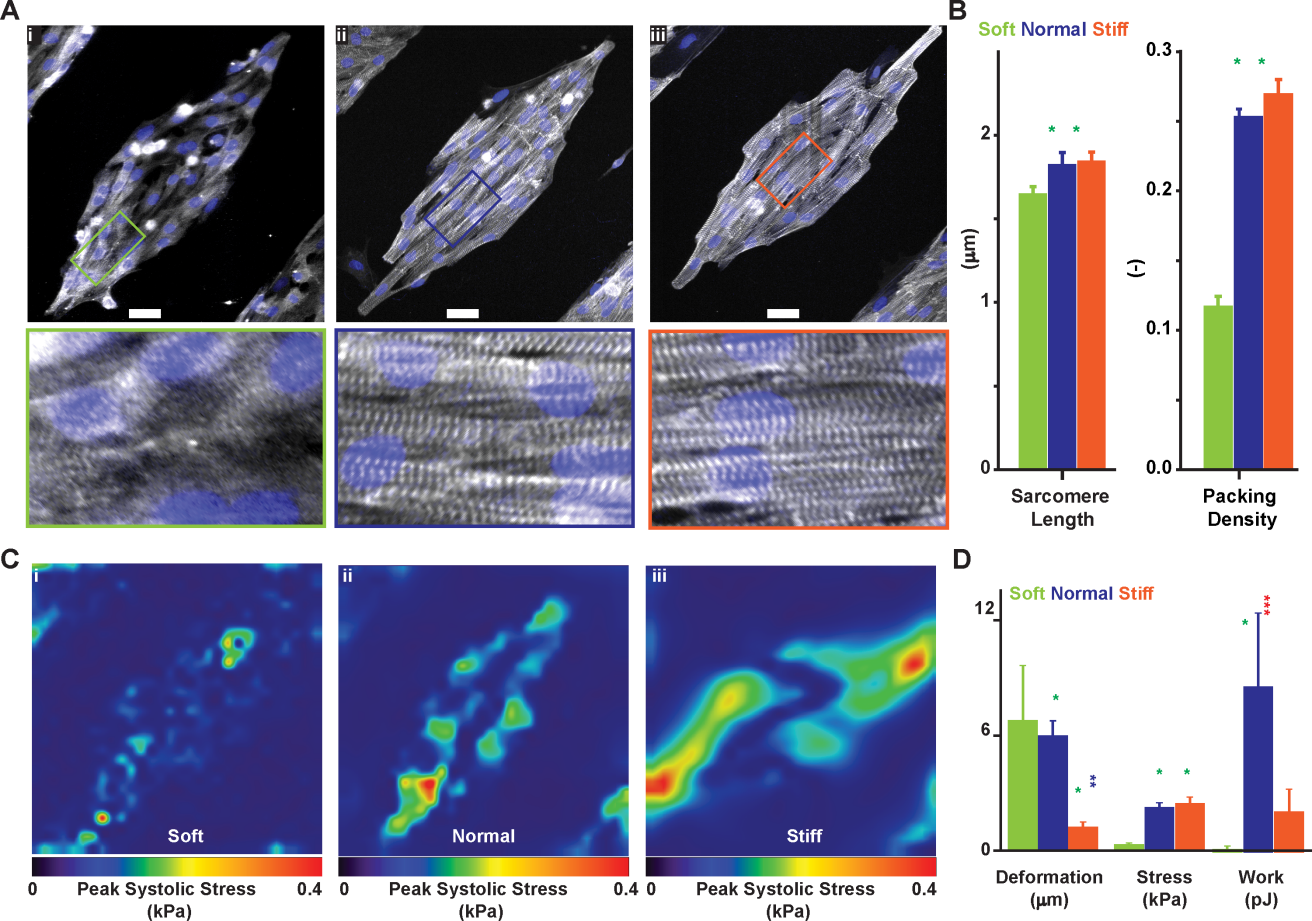
Contractile structure and function of cardiac tissues engineered on gels of varying stiffness. Using α-actinin immunographs from NRVM engineered on soft (1 kPa, A-i), normal (13 kPa, A-ii), and stiff (90 kPa, A-iii) gels we assessed differences in sarcomere length (SL) and sarcomere packing density (SPD, B). Scale bar 50 μm. Further, we performed TFM on diamond-shaped NRVM tissues engineered on soft (C-i), normal (C-ii), and stiff (C-iii) gels (peak systolic stress shown) to calculate (D) maximum deformation, peak stress, and total contractile work (strain energy). Results are given as mean ± s.e.m. and N = 3, 4, and 7 for soft, normal, and stiff gels respectively. The symbol * implies significant differences (p < 0.05) compared with the group of the same color.

A number of important phenotypic differences occur during cardiomyocytes maturation, including the expression of different transcription factors, ion channels, and contractile proteins; the appearance of physiologically-relevant structures such as t-tubules; and the progressive assembly of the contractile cytoskeleton [4]. Specifically, since the self-assembly process that leads to the formation of a mature contractile apparatus is well-conserved across species, it can be used to characterize the phenotypic maturation of cardiomyocytes [19]. We previously showed that isolated myocytes cultured on substrates with normal physiological stiffness exhibited more organized sarcomeres and exerted greater contractile stress than cells seeded on softer and stiffer substrates [5, 7, 8]. Here, we asked whether this relationship is maintained at the tissue level. Three days after seeding, we fixed and stained NRVM mTissues cultured in soft (1 kPa, Fig 2A, panel i), normal (13 kPa, Fig 2A, panel ii), and stiff (90kPa, Fig 2A, panel iii) gels using an antibody against sarcomeric α-actinin. As the cytoskeleton matures,α-actinin progressively localizes at the sarcomere edges, and we can use its spatial distribution to gauge structural maturation [20]. To achieve this, we imaged the NRVM preparations and assayed the degree of structural organization using a previously validated image processing software [19] that calculates the average sarcomere length and the sarcomere packing density, a number ranging from 0 to 1 as the regular lattice of striations appear (Fig 2B). Sarcomeres in NRVM mTissues engineered on soft substrates were 1.65±0.01 μm long (mean ± s.e.m), a significant ~10% shorter (p<0.001, N = 6) than in tissues engineered on normal physiological (1.82±0.02 μm) and stiff (1.84±0.02 μm) substrates, respectively. At the same time, sarcomeres were significantly less periodically organized on soft substrates than on stiffer substrates. We measured sarcomeric packing density [19] values of 0.11±0.002, 0.25±0.003, 0.27±0.02 (mean±s.e.m.) from NRVM on soft, normal, and stiff substrates, respectively (p<0.001, N = 6). Together, these results support the notion that, even at the tissue level, NRVM cultured on substrates with stiffness similar to the fetal heart possess an immature contractile cytoskeleton [5, 7, 8].

Further, we measured bead displacement in the mini tissue TFM platform and calculated contractile stress and work output in NRVM tissues engineered on soft (Fig 2C, panel i), normal physiological (Fig 2C, panel ii), and stiff (Fig 2C, panel iii) gels. We found that tissues on softer substrates generated significantly less contractile stress while inducing a significantly larger bead displacement than tissues engineered on stiffer substrates (p<0.05, N = 3, 4, and 7 for soft, normal, and stiff gels respectively–Fig 2D). Specifically, peak systolic stress values, averaged over five consecutive beats, were 0.30±0.05, 2.16±0.07, and 2.37±0.11 kPa (mean±s.e.m.) for soft, normal, and stiff substrates, respectively. At the same time, peak displacement values were 6.71±1.64, 5.94±0.21, and 1.22±0.08 μm for soft, normal physiological, and stiff substrates, respectively. Consistently, NRVM tissues engineered on substrates of normal physiological stiffness exhibited the largest amount of contractile work, as shown by strain energy values of 0.08±0.10, 8.86±1.04, and 2.02±0.43 pJ for soft, normal, and stiff substrates, respectively. In good agreement with the single cell and cell pair data, these results support the notion that tissues contract more strongly when engineered on gels of normal physiological stiffness [5, 7, 8].

### The metabolic profile of NRVM on substrates of different stiffness

**Fig 3:**
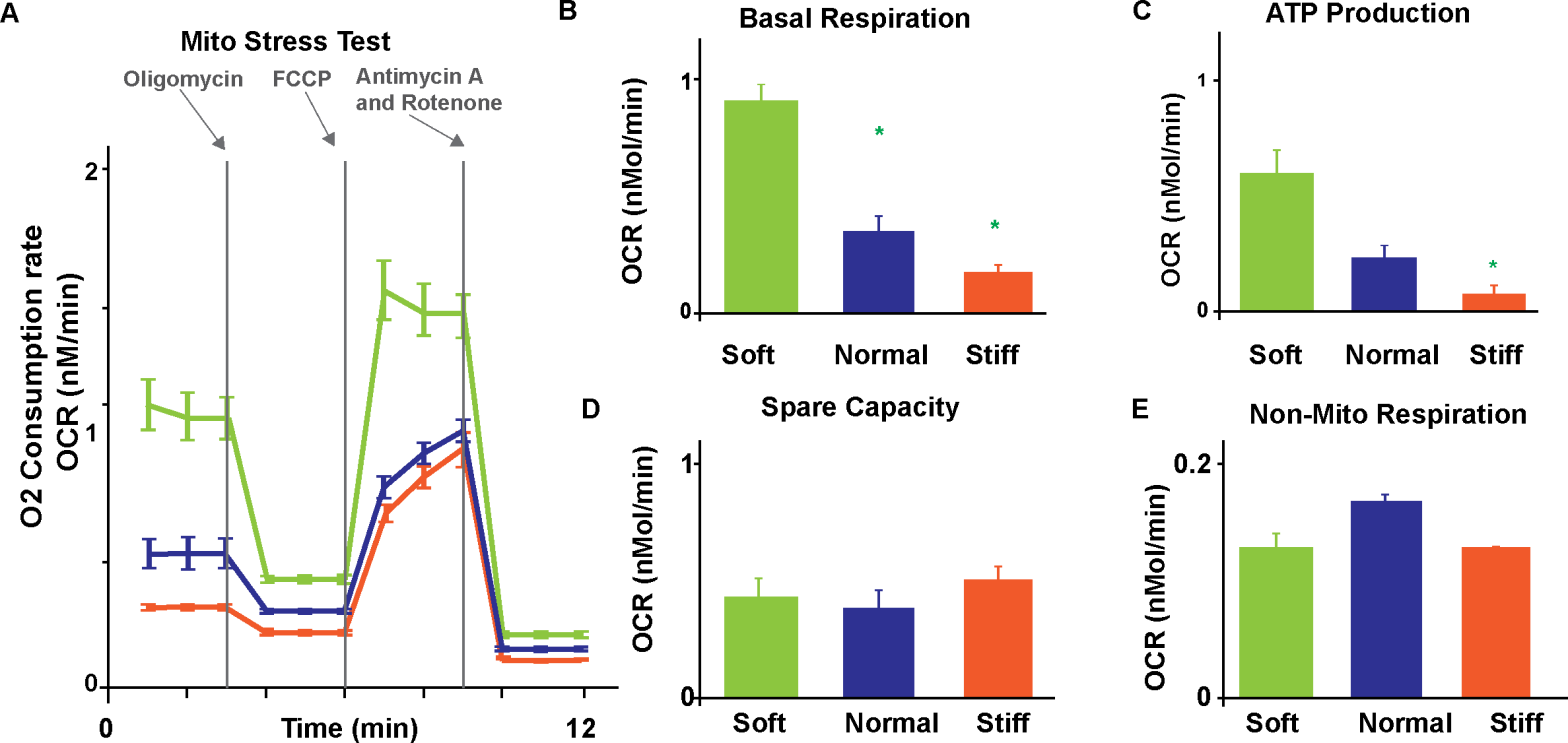
Metabolic differences in engineered cardiac tissues on gels of different stiffness. (A) We measured oxygen consumption rates of NRVM cultured on soft (1 kPa), normal (13 kPa), and stiff (90 kPa) gels during a standard mitochondria stress test. We assessed basal respiration rate (B), ATP production (C), spare respiratory capacity (D), and nonmitochondrial respiration (E) for each substrate. Results are given as mean ± s.e.m. with sample size equal to 12, 15, and 22 for soft, normal, and stiff gels, respectively. The symbol * implies significant differences (p < 0.05) compared with the group of the same color.

Heart failure with preserved ejection fraction, valve disease, diabetes, and even normal aging have all been associated with stiffer myocardium [21–24]. Importantly, as their microenvironment stiffens, cardiomyocytes adapt not only their structural and functional properties but also their metabolic profile suggesting substrate stiffness modulates the coupling between contractile work and energetic requirements [15]. To elucidate this phenomenon and to demonstrate the compatibility of our platform with existing high-throughput assays, we cast soft, normal, and stiff gels inside the 24-well plate of the Seahorse, an industry-standard flux analyzer [25]. We then performed a standard XF Cell Mito stress test assay to measure the oxygen consumption rate (OCR) in NRVM at baseline and after injection of compounds with known mitochondrial activity (Fig 3A). We first added oligomycin (2 mM) to inhibit ATP synthesis; we then added carbonyl cyanide-4-(trifluoromethoxy) phenylhydrazone (FCCP, 1 mM) to boost mitochondria-based ATP production; and finally added both antimycin A (1 mM) and rotenone (1 mM) to abolish mitochondrial respiration completely [13]. By comparing OCR values measured after drug injection and at baseline, we observed that NRVM on soft substrates had a significantly higher basal respiration (Fig 3B) and ATP production capacity (Fig 3C) than cells on normal physiological and stiff substrates (p<0.05, N>12 tissues). Specifically, in NRVM seeded on soft, normal, and stiff gels, we measured OCR values of 942.39±70.25, 373.38±66.24, and 192.51±7.02 pMol/min for basal respiration (mean±s.e.m); and 593.40±101.51, 225.27±56.74, and 82.41±7.02 pMol/min for ATP production, respectively. Independent of substrate stiffness, NRVM had similar spare capacity (Fig 3D) and relied minimally on glycolysis (Fig 3E). Specifically, in NRVM cultured on soft, normal physiological, and stiff substrates we measured OCR values of 420.76±83.60, 377.04±81.83, 508.19±57.08 pMol/min for spare capacity; and 132.47±12.30, 172.27±5.92, and 133.39±1.16 pMol/min for non-mitochondrial respiration, respectively. Together, our data show that NRVM on soft microenvironments contract less strongly and less efficiently than on stiffer substrates suggesting myocytes invest the additional available ATP in alternative processes such as cytoskeleton maturation.

## Discussion

We presented an alternative quantitative method to assess contractile structure and function at the tissue level in cardiac preparations. Our platform offers important advantages over existing alternatives. First, our technique makes it possible to use the same assessment technique, namely traction force microscopy, to study the contractile properties of cardiomyocytes in preparations ranging from single cells, to cell-pairs, to tissues [5, 7, 8]. This requirement is important for future sound experimental and theoretical investigations into the scaling law of muscle mechanics [26–31]. Second, engineering diamond-shaped mTissue requires up to two orders of magnitude fewer cells than other 2D or 3D cardiac MPS: an important aspect to consider when using rare or costly cells such as patient-specific hiPS cells [13, 14, 32, 33]. Third, the reduced cell count and the planar design of the assay are conducive to facilitate scaling and microfluidic integration for organ-linking applications [34–37]. Finally, depending on the quality of the preparation and the cells, our TFM-based platform offers a high density of diamond-shaped mini tissues (2 tissues/mm^2^) that can be addressed individually along the diagonal of the field of view of a typical 20x objective. A limitation worth mentioning is that, consistent with our experience with TFM in single cells and cell pairs, stamping of gels with stiffness less than five kPa remains difficult. Therefore, a better stamping technique might be needed to obtain the larger sample sizes needed to tease apart smaller contractile differences in cells seeded on substrates with stiffness values in the fetal range (e.g., 1, 3, and 5 kPa).

**Fig 4:**
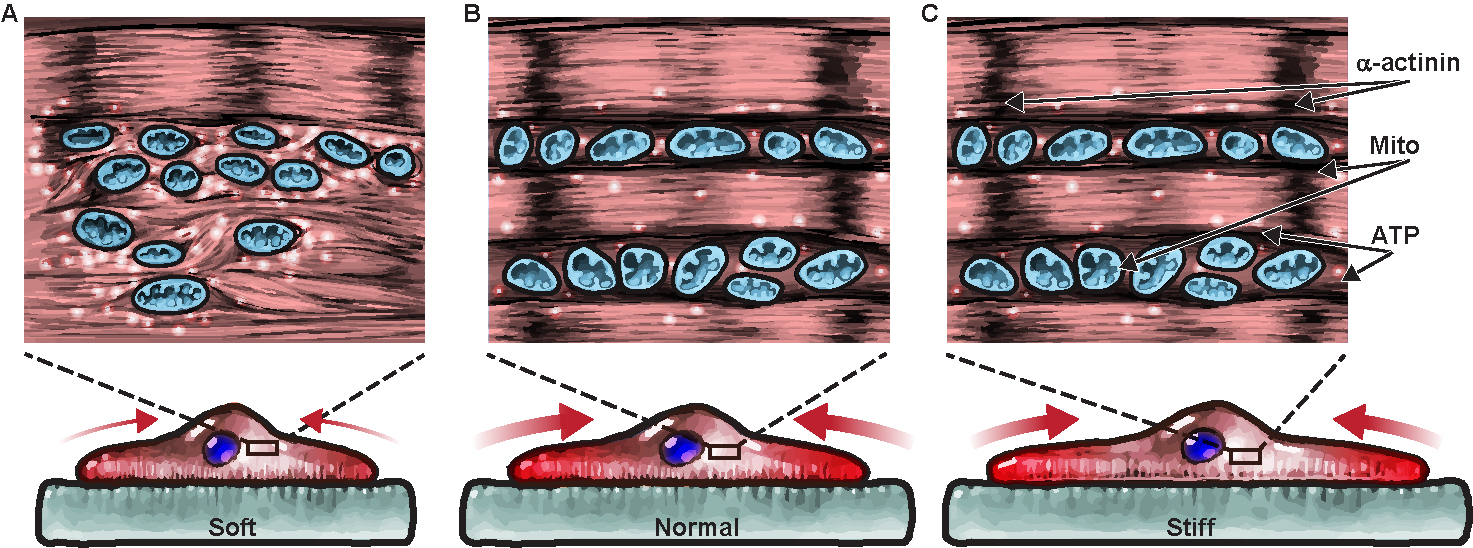
Cardiomyocytes operate optimally on substrates of physiological stiffness possibly due to a link between metabolism and contractile structure and function. Based on our data, we speculated that substrate stiffness might regulate the balance of energy production and utilization in cardiac tissues as follows. Cardiomyocytes on soft gels (A) need abundant ATP (white spheres) derived by mitochondria (orange organelles) to promote the contraction of sarcomeres (Z-disks in black) via cross-bridge cycling (brown lines). This might lead to very inefficient mechanical work as only a small stress (red arrows) is required to deform the soft gel substantially. Conversely, NRVM cultured on normal (B) and stiff (C) substrates have well organized contractile cytoskeletons and need a limited amount of ATP to fuel sarcomere contraction. Since the same amount of contractile force causes a smaller displacement of stiffer gels, the product between force and displacement (work) is maximum on gels of physiological stiffness.

As a proof-of-concept study, we combined our mTissue TFM platform with an industry-standard high-throughput metabolic flux analyzers to investigate the coupling between mechanotransduction, contractility, and metabolism. We speculate (Fig 4) that myocytes on soft substrate might require a larger amount of ATPs than cells on stiffer substrates, as they must complete the assembly of the contractile cytoskeleton in addition to performing their contractile activity. We showed that NRVM on gels with normal physiological stiffness reached an optimum coupling between energy production and energy consumption; that is, cells on substrates mimicking physiological stiffness needed the minimum amount of ATP to generate the maximum amount of work. On stiffer substrates, ATP levels and the cell-generated stress did not change leading to an overall reduction in displacement and work. Conversely, on softer substrates, NRVM had the largest amount of ATP available to generate the minimum amount of work via an immature contractile apparatus [4, 19]. An efficiency metric can be used to characterize the coupling between energy production (metabolism) and energy utilization (sarcomerogenesis and contractility) in cardiomyocytes; that is, the ratio between the contractile work done by the cells and the metabolic energy provided by the mitochondria [38]. To compare contractile work and oxygen consumption rates, we converted [pMol/min] of ATP into the corresponding [pJ/beat] values using the following assumptions. First, NRVM beat 120 times in a minute under the 2Hz pacing. Second, the surface area of a Seahorse well is 550x larger than a diamond tissue surface area, leading to a similar increase in cell number [16]. And third, a Mole of ATP provides ~29 kJ of energy [39]. With these approximations, our results demonstrate that tissues engineered on substrates of physiological stiffness convert metabolic energy into contractile work ~2x and ~200x better than they do on stiffer and softer substrates, respectively. One limitation of this analysis is that we only partially control for the cell beating frequency. In fact, intrinsic beating activity in primary [40–42] and stem cell-derived cardiomyocytes [43, 44] changed as much as 20% and 300% as a function of substrate stiffness and the type of preparation. Here, we minimized the difference between spontaneous metabolism and stimulated contractility by choosing the smallest pacing frequency (2 Hz) that overdrove the NRVM intrinsic activity. To overcome this limitation in future efforts, we envision using optical pacing of cells expressing light-sensitive ion channels [45].

In conclusion, the tissue-level assay presented here completes a consistent suite of TFM-based assays that can enable thorough multi-scale studies of the coupling between metabolism, mechanotransduction, and contractility in primary and stem cell-derived myocytes [5–8]. Two recent studies from Prof. McCain’s group addressed similar topics: tissue-level cardiac TFM [46] and NRMV metabolism as a function of substrate stiffness [47]. While these studies are consistent with our findings, a few important differences exist. For example, they engineered NRVM in square-shaped tissues on normal and stiff gels using fibronectin and laminin cues [46] we used larger, diamond-shaped Fibronectin-only cues to improve cellular alignment in mTissues cultured on soft, normal, and stiff gels. Consistent with our study, stiffening of the substrate caused a sharp decline in cell-induced deformation and contractile work in engineered tissues, independently from the chosen ECM ligand. Similarly, to obtain substrates of pathological stiffness, we had to cast hydrogels directly into the very small wells of the Seahorse plate and could only culture randomly-oriented NRVM (1, 13, and 90 kPa). Instead, the metabolic difference between randomly-oriented and laminar tissues was studied [47] by engineering cells on transferrable elastomeric substrates prepared in a range of stiffness that greatly exceeds the pathophysiological range (1, 27, and 2700 kPa). In contrast to our findings, a reduced metabolic activity was observed on NRVM cultured on soft substrates under both laminar and non-laminar conditions. We attribute this difference to the distinct chemical and viscoelastic properties of elastomers and hydrogels that may influence the density of printed ECM proteins and cellular behavior [48, 49]. We believe engineered cardiac tissues will become progressively more important as in studying the energetic coupling underpinning cardiac development and homeostasis [12, 15], and might inform the design of better differentiation and maturation protocols for hiPS-derived cardiomyocytes [4, 19].

## Materials and methods

### Cardiac myocyte harvest, seeding, and culture

All animal procedures conducted in this study were approved by the Harvard University Animal Care and Use Committee. Cardiac myocytes were extracted from neonatal rat ventricles using a previously described method [7, 8]. Left ventricle tissue was isolated from neonate (p2) Sprague-Dawley rats (Charles River) that were sacrificed after ethanol-based anesthetization. Cells were isolated using overnight trypsinization at 4° C and serial collagenase digestions at room temperature. A highly pure cardiomyocyte population was obtained by differential adhesion using two pre-plating steps. Cardiomyocytes were cultured on engineered substrates in 6-well plates at a density of 100,000 cells/cm^2^ in Medium 199 supplemented with 10% heat-inactivated fetal bovine serum (FBS, Invitrogen, Carlsbad, CA), 10 mM HEPES, 0.1 mM MEM non-essential amino acids, 20 mM glucose, 2 mM L-glutamine, 1.5 μM vitamin B-12, and 50 U/mL penicillin for 1 day. The serum concentration was reduced to 2% after 24 hr to minimize proliferation of the small pool of fibroblast present in the culture. Cells were cultured for four days before conducting the experiments.

### Gel fabrication and microcontact printing

Polyacrylamide gel substrates were fabricated and characterized as previously described in details [7, 8]. Specifically, three groups of gels were created by varying the acrylamide/bis composition. Soft gels were obtained mixing 5/0.1% acrylamide/bis components and had a Young modulus of 0.9±0.02 kPa (nominal stiffness 1 kPa). Normal gels were obtained mixing 7.5/0.3% acrylamide/bis and had a Young modulus of 13±0.1 kPa (nominal stiffness 13 kPa). Stiff gels were obtained mixing 12/0.6% acrylamide/bis and had a Young modulus of 90±1.5 kPa (nominal stiffness 90 kPa). Furthermore, streptavidin-acrylamide and fluorescent beads (200 n m) were added to a final concentration (by volume) of 1:5 and 1:100, respectively to permit binding biotinylated-fibronectin (Sulfo-NHS-LC-Biotin, Pierce). Gels polymerized while sandwiched between glutaraldehyde-activated and inactivated coverslips before drying the gel surface (37 °C for 10 minutes) and microcontact printing with biotinylated FN.

Photo- and soft-lithography were conducted as previously described in details [7, 8, 10]. Specifically, a photolithographic mask carrying the desired diamond and frame features was designed in AutoCAD (Autodesk Inc.) and manufactured at the Center for Nanoscale Systems at Harvard University. Silicon molds to fabricate polydimethylsiloxane (PDMS, Sylgard 184, Dow Corning) stamps were prepared by spin-coating SU-8 2002 photoresist (MicroChem Corp) on silicon wafers (Wafer World) that were exposed to UV light through the photoligraphic mask in a mask aligner (ABM Inc.)and then developed in propylene-glycol-methyl-ether-acetate to dissolve the masked regions. The resulting PDMS stamps were coated with 200 μg/mL biotinylated FN and incubated for 1 hr before stamping on pre-dried streptavidin-doped polyacrylamide gels.

### Contractility assay and electrical field stimulation

Traction force microscopy experiments were conducted as previously described [7, 8, 10]. On day 4 of culture, cardiac mTissues in modified Tyrode’s solution (1.8 mM CaCl_2_, 5 mM glucose, 5 mM HEPES, 1 mM MgCl_2_, 5.4 mM KCl, 135 mM NaCl, 0.33 mM NaH_2_PO_4_, pH 7.4) were imaged on an environmental controlled line scanning confocal microscope (Zeiss LSM510) using a 40X air objective (with a 0.5x zoom) or a 20x objective to ensure the full diamond-shaped tissue fit within the diagonal of the field of view. Movies of contracting myocytes and bead displacement were imaged at 33 Hz with both brightfield, and 488 laser excitation and recordings were performed over multiple [6–10] contractile cycles. Differently, than single-cell or cell-pair assays, mTissues could be electrically paced to control the beating rate using two custom-made platinum electrodes inserted onto the lid of a 35mm petri dish and connected to an external field stimulator (Myopacer, IonOptix Corp., Milton, MA). Recordings were conducted at 2 Hz and using voltages between 8 to 11 V that reliably overdrove spontaneous beating in the mTissues.

### Contractility analysis

The methods used to acquire displacement and traction stress vectors from images of bead displacement have been previously described [7, 18]. Briefly, a displacement field was determined by comparing bead images throughout the contraction cycle to the bead image at diastole. The traction stress field was then calculated from the displacement map using constrained Fourier transform traction cytometry. Importantly, we used the bright field video acquired together with the bead displacement to define the boundary of the mTissue in calculating the traction field. Moreover, the contractile traction force field was calculated from the displacement field using the Boussinesq solution and assuming the substrate as an elastic half-space with known mechanical properties (stiffness of 1, 13, or 90 kPa and Poisson ratio assumed to be 0.5). Notably, this particular assumption, important in accelerating the calculations needed to obtain a traction field, becomes less accurate as the size of the contractile elements grow larger than the thickness of the tissue [50–52]. We used a more accurate boundary element method [18] that admits a Green function characteristic of gels with finite thickness [53] to estimate the error and found that peak systolic stress and contractile work are slightly overestimated (1–15%) with the simple FTTC method. At the same time the more accurate analysis took ~10 hr/frame on a powerful workstation computer to vs ~3 min/frame in the case of the simpler FTTC analysis. Since we processed 300 frames worth of gel deformation per mTissues, we utilized the faster, if slightly less accurate, analysis in this study.

### Immunostaining and structure determination

Image analysis and processing were performed as previously described [5]. After the contractility assay, samples were incubated for 15 minutes in 4% Paraformaldehyde (PFA) and 0.5μl/ml of TritonX-100 in PBS at 37°C before washing in PBS and incubation with 200 μl primary antibody solution containing 1 DAPI, 1 μl Alexa Fluor633-conjugated Phalloidin (Invitrogen, Carlsbad, CA), 1 μl polyclonal anti-human fibronectin antibody (F2648, Sigma-Aldrich, St. Louis, MO), and 1 μl monoclonal anti-sarcomeric α-actinin for 1 hour at room temperature. Samples were then washed three times in PBS and incubated for 1 hour with a goat anti-rabbit Alexa Fluor-546 antibody and a goat anti-mouse Alexa Fluor-488 antibody (Invitrogen, 1:200 dilution). The samples were washed three times again in PBS and mounted on microscope glass slides in prolongGold (Invitrogen) that cured for at least 48 hr before fluorescent imaging on a Zeiss LSM 510 confocal microscope. Sarcomere length and sarcomere packing density were determined using a Fourier-based method previously described [19].

### Seahorse metabolic measurements

Cellular metabolism was measured using a Seahorse Bioscience XFe 24 Extracellular Flux Analyzer (North Billerica, MA). Polyacrylamide gels were pipetted into the wells (10 mL/well) of a standard XF24 microplate (Seahorse Bioscience, North Billerica, MA) and allowed to crosslink before rinsing with PBS. Plates were stored at 4 °C until cell seeding with neonatal rat ventricular myocytes (100 000 cells/100 mL/well). After seeding, plates were left in the laminar flow hood for 30 min to minimize edge effects before transfer to a 37 °C, 5% CO_2_ incubator. After two hours, 500 mL of media was added to each well and media exchanges were performed as previously described above. After four days in culture, culture media was replaced with XF Assay Medium (Seahorse Bioscience, North Billerica, MA) supplemented with 20 mM glucose and plates were incubated at a 37 C for 1 h. The proprietary hydrated cartridge was loaded with 2 mM oligomycin, 1 mM carbonyl cyanide-4-(trifluoromethoxy) phenylhydrazone (FCCP), and 1 mM antimycin A and 1 mM rotenone from the XF Cell Mito Stress Test Kit (Seahorse Bioscience, North Billerica, MA). The cartridge and cell plate were then inserted into the Seahorse XFe 24 Extracellular Flux Analyzer (North Billerica, MA), which made three measurements of baseline oxygen consumption rate (OCR) and three OCR measurement after injection of each of the drugs listed above. Data from multiple wells across three different neonatal rat ventricular myocyte harvest cycles were collected and used for analysis.

### Statistical analysis

Through the text, our results are presented as mean ± standard error of the mean (s.e.m.). After verifying that the data points were normally distributed (Shapiro-Wilkinson test), statistical comparisons were conducted among the various group using 1-way ANOVA test followed by Tukey pairwise comparisons. All analyses were conducted using SigmaPlot (Systat Software, Inc.–CA, USA). The symbol * was used to denote statistically significant difference characterized by a *p*-value smaller than 0.05.

## Supporting information

S1 Checklist The ARRIVE guidelines checklist. (PDF)
